# Identification and characterisation of anti-IL-13 inhibitory single domain antibodies provides new insights into receptor selectivity and attractive opportunities for drug discovery

**DOI:** 10.3389/fimmu.2023.1216967

**Published:** 2023-07-06

**Authors:** Kayleigh Walker, Roberta Baravalle, Rachel Holyfield, Jacqueline Kalms, Helena Wright, Chitra Seewooruthun, Frederick W. Muskett, Anthony Scott-Tucker, Andy Merritt, Alistair Henry, Alastair D. G. Lawson, Gareth Hall, Christine Prosser, Mark D. Carr

**Affiliations:** ^1^ Leicester Institute of Structural and Chemical Biology, and Department of Molecular and Cell Biology, University of Leicester, Leicester, United Kingdom; ^2^ UCB Biopharma, UCB Pharma, Slough, United Kingdom; ^3^ LifeArc, Centre for Therapeutics Discovery, Stevenage Bioscience Catalyst, Stevenage, United Kingdom

**Keywords:** interleukin-13, single domain antibodies, VHH, receptor signalling, receptor selectivity, allosteric regulation

## Abstract

Interleukin-13 (IL-13) is a cytokine involved in T-cell immune responses and is a well validated therapeutic target for the treatment of asthma, along with other allergic and inflammatory diseases. IL-13 signals through a ternary signalling complex formed with the receptors IL-13Rα1 and IL-4Rα. This complex is assembled by IL-13 initially binding IL-13Rα1, followed by association of the binary IL-13:IL-13Rα1 complex with IL-4Rα. The receptors are shared with IL-4, but IL-4 initially binds IL-4Rα. Here we report the identification and characterisation of a diverse panel of single-domain antibodies (VHHs) that bind to IL-13 (K_D_ 40 nM-5.5 μM) and inhibit downstream IL-13 signalling (IC_50_ 0.2-53.8 μM). NMR mapping showed that the VHHs recognise a number of epitopes on IL-13, including previously unknown allosteric sites. Further NMR investigation of VHH204 bound to IL-13 revealed a novel allosteric mechanism of inhibition, with the antibody stabilising IL-13 in a conformation incompatible with receptor binding. This also led to the identification of a conformational equilibrium for free IL-13, providing insights into differing receptor signalling complex assembly seen for IL-13 compared to IL-4, with formation of the IL-13:IL-13Rα1 complex required to stabilise IL-13 in a conformation with high affinity for IL-4Rα. These findings highlight new opportunities for therapeutic targeting of IL-13 and we report a successful ^19^F fragment screen of the IL-13:VHH204 complex, including binding sites identified for several hits. To our knowledge, these ^19^F containing fragments represent the first small-molecules shown to bind to IL-13 and could provide starting points for a small-molecule drug discovery programme.

## Introduction

Interleukin-13 (IL-13) is a Th2-type cytokine exhibiting both proinflammatory and anti-inflammatory effects that is produced by several cell types, including activated T-helper type 2 cells, basophils, eosinophils and mast cells (1–3). It displays a pleiotropic behaviour by eliciting different cell types, including B-cells, fibroblasts, macrophages and endothelial cells (4). While IL-13 is a key mediator in the onset of asthma, by playing an active role in IgE production, mucus hypersecretion, airway fibrosis and hyperreactivity to inhaled spasmogens (5–7), it also is the dominant cytokine in the induction of tissue fibrosis associated with chronic inflammation (8). In addition, IL-13 has crucial immunosuppressive and anti-inflammatory functions by inhibiting the production and release of other proinflammatory cytokines, such as IL-1, IL-6 and IL-12, as well as downregulating inflammatory mediators such as leukotrienes and prostaglandins (9). IL-13 shares 25% amino acid sequence homology with interleukin-4 (IL-4), and their close functional relationship is evidenced by the sharing of two cell surface receptors. IL-4 signalling is mediated *via* the assembly of either type-I receptor complexes, including IL-4Rα and the common gamma-chain (γ_c_), or *via* the formation of type II receptor complexes with IL-4Rα and IL-13Rα1, which is also the functional receptor complex for IL-13 (10). IL-4 and IL-13 have distinct sequential signalling complex formation. IL-4 initially interacts with IL-4Rα (K_D_ 1 nM) followed by binding of this binary complex to either γ_c_ (K_D_ 559 nM) or IL-13Rα1 (K_D_ 487 nM). In contrast, IL-13 has negligible affinity for IL-4Rα alone and must first bind to IL-13Rα1 (K_D_ 30 nM), which greatly enhances the interaction with IL-4Rα (K_D_ 20 nM) (11).

IL-13 and IL-4 are proto-typical four-helix bundle, short-chain cytokines, with an up-up-down-down topology of the helices (12–14). There are a few important structural differences between IL-13 and IL-4, including an additional disulphide bond between helices A and D in IL-4 and an extended helix C. In the formation of type II receptor signalling complexes both IL-4 and IL-13 interact with IL-13Rα1 *via* a hydrophobic cleft between helices A and D, which contains different amino acids in the two cytokines but retains the same surface shape and hydrophobicity (11). There are two charged residues in IL-4 (E9 and R88), which are essential and form key interactions with IL-4Rα. These residues are conserved in IL-13 (E12 and R65), however, the surrounding receptor binding surface of IL-13 differs from IL-4. The IL-4Rα binding site of IL-4 is predominantly positively charged with the corresponding surface of IL-4Rα negatively charged (15), but this charge complementarity is not conserved in the interface between IL-13 and IL-4Rα, which in part probably accounts for the negligible affinity between free IL-13 and IL-4Rα.

Currently, there are several anti-IL-13 therapeutic antibodies available for the treatment of allergic and inflammatory diseases (16–18). The IL-13 and IL-4 signalling pathways have also been implicated in cancer biology (19), which further highlights IL-13 as a well-validated therapeutic target. Here, we report the results of a pioneering antibody-assisted drug discovery approach applied to IL-13, which identified attractive new options for small molecule drug discovery, together with providing new insights into receptor selectivity and allosteric regulation of IL-13 (20). We describe the identification and characterisation of a diverse panel of inhibitory llama single-domain antibodies (VHHs) targeting IL-13, including NMR-based mapping of the antibody epitopes, which led to the identification of several previously unknown allosteric regulatory sites on IL-13. The allosteric acting VHH204 was found to have an interesting mechanism of inhibition, involving stabilisation of a conformation of IL-13 unable to bind IL-13Rα1 (receptor-incompetent state). This led to the identification of a functionally important conformational equilibrium present in free IL-13, which provides a molecular basis for the differing receptor selectivity and staged ternary signalling complex assembly seen for IL-13 and IL-4. To assess the potential to identify small-molecule fragments that bound specifically to the receptor-incompetent conformation of IL-13 we screened a ^19^F fragment library against the IL-13:VHH204 complex. The screening of small molecule modulators against cytokines has been previously implemented against IL-2 (21), whereby the small molecule inhibitors targeted allosteric sites that are inherently adaptive between the free- and receptor-bound IL-2, and also induced structural features incompatible to IL-2:IL-2Rα complex formation (22). Similarly, in this study the ^19^F fragment screen against the IL-13:VHH204 complex identified a number of hits that were shown to bind to several functionally significant regions on IL-13, that could potentially be developed to stabilise the receptor-incompetent state of the cytokine.

## Materials and methods

### Protein expression and purification

The coding region for human IL-13 (1–113) was cloned into pET3a(+). IL-13 was expressed as inclusion bodies in BL21 (DE3) pLysS *E. coli* cells (Novagen). For NMR studies uniformly ^15^N and ^15^N/^13^C labelled IL-13 was expressed in BL21 (DE3) pLysS *E. coli* cells cultured in modified Spizizen’s minimal media (23) containing ^15^N-NH_4_Cl and ^13^C-glucose. For non-isotopically labelled IL-13, cells were cultured in LB media, at 37 °C, and protein expression induced with 0.5 mM IPTG at an optical density of 0.7 at 600 nm. The cells were cultured for 16 h before harvesting by centrifugation. Insoluble IL-13 was refolded and purified by optimising the procedure described by Moy et al., 2001 (13). Cell pellets were resuspended in 50 mM Tris-HCl, pH 8.0, supplemented with cOmplete protease inhibitors (Roche), 1 mM MgCl_2_, benzonase (MilliporeSigma) and 0.5 mg/mL lysozyme, and lysed by cell disruption (Constant Systems) at 30 kpsi before inclusion bodies were collected by centrifugation. Inclusion bodies containing IL-13 were washed twice with 100 mM Tris-HCl, pH 7.8, 5 mM DTT, 5 mM EDTA, 2 M urea, 1.0% v/v Triton X-100 and once in the same buffer without detergent and urea. Following washing, inclusion bodies were resolubilised in 6 M guanidine-HCl, 50 mM Tris-HCl, pH 8.5, 1 mM EDTA and 20 mM DTT at 2 mg/mL. Resolubilised IL-13 was refolded by dropwise dilution into refolding buffer (50 mM Tris-HCl, pH 8.2, 100 mM NaCl, 3 M guanidine-HCl, 1 mM oxidised glutathione) with a dilution of 1:20. IL-13 was refolded at room temperature for 24 h. The refolding mixture was concentrated by tangential flow filtration (Sartorius) and then dialysed into 25 mM Tris-HCl, pH 7.5, 100 mM NaCl, or NMR buffer (25 mM sodium phosphate pH 6.0, 100 mM NaCl), prior to purification by size exclusion chromatography using a Superdex-75 column (GE Healthcare).

VHH coding sequences were cloned into pET21a(+) with an N-terminal hexa-histidine tag. VHHs were expressed as inclusion bodies in BL21 (DE3) *E. coli* cells (Novagen). Cells were cultured at 37°C in LB media and protein expression was induced with 0.5 mM IPTG at an optical density of 0.7 at 600 nm. The cells were then cultured overnight before harvesting by centrifugation. Cell pellets were resuspended in 50 mM Tris-HCl, pH 8.0, supplemented with cOmplete protease inhibitor (Roche), 1 mM MgCl_2_, benzonase (MilliporeSigma) and 0.5 mg/mL lysozyme and lysed by cell disruption at 27 kpsi before inclusion bodies were collected by centrifugation. The inclusion bodies were washed twice with 100 mM Tris-HCl, pH 7.8, 5 mM EDTA, 2 M urea, 1.0% v/v Triton-X 100 and 5 mM DTT, and once in the same buffer without detergent and urea. Washed inclusion bodies were resolubilised in 6 M guanidine-HCl and 2 mM DTT at 0.5 mg/mL and refolded by dialysis. Resolubilised inclusion bodies were dialysed twice against a 10X volume of 50 mM Tris-HCl, pH 8.5, 1 M guanidine-HCl, 0.2 mM oxidised glutathione and 1 mM reduced glutathione, and then twice against a 10X volume of 1X PBS, pH 7.4. The refolded N-His tagged VHHs were initially purified by affinity chromatography on a Ni-NTA superflow column (QIAGEN). The column was washed with 5 column volumes of 1X PBS, pH 7.4, and 20 mM imidazole, followed by elution of the VHHs over a 10-column volume gradient of imidazole from 20 to 500 mM. Final purification of VHHs was performed by size exclusion chromatography using a Superdex-75 column (GE Healthcare) previously equilibrated in 25 mM Tris-HCl pH 7.5 and 100 mM NaCl or NMR buffer (25 mM sodium phosphate pH 6.0, 100 mM NaCl).

### VHH phage display biopanning of IL-13

In order to perform phage display biopanning of IL-13, purified IL-13 was biotinylated on its positively charged surface residues using a Lightning-Link Rapid Biotin Conjugation Kit (Type B), following manufacturer’s instructions (Innova Biosciences). A naïve llama VHH phage library, provided by UCB Biopharma, was used to perform enrichment of phage that bound to IL-13, using a protocol adapted from Wilkes et al., 2020 (24). 500 nM biotinylated IL-13 was immobilised on a Nunc MaxiSorp ELISA plate (Thermo Fisher) coated with 5 μg/mL neutravidin (Thermo Fisher) in 1X PBS, pH 7.4, for round one (R1) of biopanning and 5 μg/mL streptavidin (Thermo Fisher) in 1X PBS, pH 7.4, for round two (R2). In parallel, neutravidin- or streptavidin-only plates were used as control. Following incubation, all unbound phage were removed with 5 to 20 washes in PBS-T (0.05% v/v Tween-20 in 1X PBS, pH 7.4) followed by two washes in 1X PBS, pH 7.4, to remove the detergent. Bound phage were eluted with 100 mM HCl and subsequently neutralised with 1 M Tris-HCl, pH 8.0.

Monoclonal rescue was performed on individual phage colonies resulting from R2 of biopanning, as previously described (24). Subsequently, monoclonal ELISA assays were performed to confirm binding of VHHs to IL-13. Briefly, 10 μg/mL biotinylated IL-13 in E-Blocking (1% w/v BSA in 1X PBS, pH 7.4) was immobilised on a Nunc MaxiSorp ELISA plate previously coated over night at 4°C with 5 μg/mL streptavidin and then blocked for 1 h with E-Blocking. In parallel, a streptavidin-only plate was used as a control. Monoclonal rescued phage were blocked with P-Blocking (2% w/v BSA and 2% w/v milk in 1X PBS, pH 7.4), added to individually washed wells and incubated for 1 h. HRP-linked anti-M13 antibody (Thermo Fisher) at a final dilution of 1:10,000 in P-Blocking was added to each washed well and incubated for 1 h. 50 μL of 1-Step Ultra TMB-ELISA substrate solution (Thermo Fisher) was added to each well and the reaction was allowed to proceed for 10 min at room temperature. The reaction was quenched by adding 50 μL/well of 2.5% w/v of sodium fluoride in water. A microplate reader (Versamax) was used to read the target absorbance at 630 nm and background at 490 nm. VHH clones that showed binding to IL-13 in the monoclonal ELISA were sequenced by Eurofins Genomics. The cDNAs of the identified unique binders were then reformatted from the phagemids into a pET21a(+) vector by Ligation-Independent Cloning (LIC) using the In-Fusion HD Cloning Kit (Takara Bio) following manufacturer’s recommendations.

### Bio-layer interferometry experiments

Binding between IL-13 and the VHHs was assessed by bio-layer interferometry using an Octet QKe system (Sartorius). Protein samples were diluted into 1X HBS-EP+ buffer (10 mM HEPES, pH 7.4, 150 mM NaCl, 3 mM EDTA and 0.005% v/v Tween-20) and experiments were carried out at 25°C, with 1,000 rpm constant shaking. Ni-NTA biosensors (Sartorius) were loaded with each N-terminally His-tagged VHH at a concentration of 100 nM until a binding response of approximately 1.5 nm was achieved. The VHHs were titrated with increasing concentrations of IL-13 for a total time of 300 s association followed by 300 s dissociation. Raw data were double referenced and aligned using the Octet Data Analysis Software (Sartorius) and analysed using Prism 7 software (GraphPad).

### In-cell activity assays

The effect of VHHs on IL-13 signalling was investigated using an in-cell activity assay in HEK-Blue IL-4/IL-13 cells (InvivoGen), specifically designed to monitor the activation of the STAT6 signalling pathway induced by IL-13 *via* the expression of a soluble reporter gene, secreted embryonic alkaline phosphatase (SEAP). Cells, maintained in a static incubator set at 37°C and 5% CO_2_ atmosphere, were cultured according to manufacturer’s recommendations in Growth Medium: Dulbecco’s Modified Eagle Medium (DMEM; Sigma Aldrich) supplemented with 10% v/v fetal bovine serum (FBS; Sigma Aldrich), 50 U/mL penicillin (Sigma Aldrich), 50 μg/mL streptomycin (Sigma Aldrich), 2 mM L-glutamine (Sigma Aldrich), 100 μg/mL normocin (InvivoGen), 10 μg/mL blasticidin (InvivoGen) and 100 μg/mL zeocin (InvivoGen). The assays were performed in Test Medium: modified Growth Medium lacking blasticidin and zeocin, and with FBS replaced with heat-inactivated FBS (SigmaAldrich). All proteins used for the experiments were diluted in Test Medium, with purified IL-13 between 0 and 100 ng/mL used to build a calibration curve reflecting cytokine activity. IL-13 at 5 ng/mL in the absence or presence of a saturating concentration of each VHH (10 times the K_D_ determined by BLI) was used to evaluate the effects of the VHHs on signalling activity. In addition, IL-13 from OriGene was used as a positive control and IL-6 from InvivoGen as a negative control. 180 μL/well of cells were seeded on a 96-well microwell plate at a cell density of 280,000 cells/mL for the assays. 20 μL protein samples (pre-incubated for 30 min at room temperature) were dispensed into each well and cells were incubated for 24 h in a static incubator set at 37°C with 5% CO_2_ atmosphere. Next, 20 μL of the cell supernatant were transferred to a new 96-well plate and 180 μL/well of QUANTI-Blue substrate (InvivoGen) pre-warmed at 37°C was added. Following a static incubation at 37°C for 10 min, to allow the SEAP to metabolise the QUANTI-Blue into a colorimetric-detectable product, the 96-well plate was transferred to a microplate reader (Versamax) to measure the absorbance at 640 nm. The assay results were analysed using Prism 7 software (GraphPad).

### NMR spectroscopy: VHH epitope mapping

For epitope mapping of VHHs, ^15^N/^1^H TROSY-HNCO (25) spectra were acquired from uniformly ^15^N/^13^C labelled IL-13 (110 μM) with a 10% molar excess of VHH in a 25 mM sodium phosphate, pH 6.0, 100 mM NaCl, 10 μM EDTA, 0.02% w/v sodium azide buffer containing 5% D_2_O/95% H_2_O. TROSY-HNCO experiments were collected using either a Bruker Avance II 800 MHz spectrometer or Bruker Avance III 600 MHz spectrometer, equipped with a cryoprobe, at 25°C with acquisition times of 80 ms in ^1^H, 21 ms in ^15^N and 25 ms in ^13^C. Non-uniform sampling (NUS) of 25% was used during data collection and datasets were reconstructed using the IST algorithm within NMRPipe (26). NMRPipe was used for data processing and spectra were analysed using NMRFAM-Sparky (27). The minimum chemical shift change for each backbone amide NMR cross peak between free and VHH-bound IL-13 was determined by calculating the lowest possible combined shift change using the following equation: $\Delta \delta =\frac{\sqrt{{\left(\Delta \delta_{HN}\right)}^{2}+{\left(\Delta \delta_{N}\cdot 0.2\right)}^{2}+{\left(\Delta \delta_{C}\cdot 0.35\right)}^{2}}}{3}$ where Δ*δ*_HN,_ Δ*δ*_N_ and Δ*δ*_C_ correspond to the difference in ^1^H, ^15^N and ^13^C chemical shifts between free and bound spectra.

NMR spectroscopy: sequence-specific backbone resonance assignments of IL-13 when bound to VHH204

Sequence-specific backbone resonance assignments for IL-13 bound to VHH204 were determined using a combination of TROSY-HNCACB, TROSY-HN(CO)CACB, TROSY-HNCA, TROSY-HN(CO)CA, TROSY-HNCO and TROSY-HSQC spectra (25). 3D NMR experiments were acquired from a 240 μM sample of uniformly ^15^N/^13^C labelled IL-13 bound to unlabelled VHH204 (10% molar excess of the VHH), in a 25 mM sodium phosphate, pH 6.0, 100 mM NaCl, 10 μM EDTA, 0.02% w/v sodium azide buffer containing 5% D_2_O/95% H_2_O. NMR experiments were collected on a Bruker Avance III 600 MHz spectrometer, equipped with a cryoprobe, at 25 °C. Typical acquisition times were 90 ms in ^1^H, 18 ms in ^15^N and 8 ms in ^13^C (25 ms for CO). Triple resonance experiments were acquired using NUS at 32% and datasets were reconstructed using the IST algorithm within NMRPipe. NMRPipe was used for data processing with the effective acquisition time in ^1^H reduced to 60 ms for the TROSY-HNCA and TROSY-HN(CO)CA experiments. Analysis of all spectra was carried out manually using NMRFAM-Sparky. For the detection of selected NOEs for IL-13 when bound to VHH204 an ^15^N/^1^H NOESY-TROSY experiment was collected from a 300 μM sample of uniformly ^15^N labelled IL-13 bound to VHH204 (10% molar excess of the VHH) under the same conditions as described above (28). The experiment was collected with acquisition times of 70 ms in direct ^1^H, 18 ms in ^15^N, and 18 ms in indirect ^1^H, with an NOE mixing time of 600 ms. NUS of 32% was used during data collection and the data were reconstructed using the IST algorithm within NMRPipe. During processing the effective acquisition time of direct ^1^H was cut back to 45 ms. Analysis of all spectra was carried out manually using NMRFAM-Sparky.

### ^19^F fragment screening of the IL-13:VHH204 complex

A library of approximately 1100 fluorine containing fragments were cocktailed into groups of 12 ensuring no overlap of ^19^F signals. Cocktails were initially prepared at a concentration of 4.2 mM in d_6_-DMSO and diluted to 800 µM in 50 mM Tris-HCl, pH 7.5, 100 mM NaCl before a final dilution to 40 µM ligand concentration (1% d_6_-DMSO) in either 50 mM Tris-HCl, pH 7.5, 100 mM NaCl containing 10% D_2_O (for control samples), or as above with the addition of 20 µM IL-13:VHH204 complex (for protein complex samples). NMR spectra were acquired at 25°C on a Bruker 600 MHz Avance Neo spectrometer fitted with a 5 mm QCI-F CryoProbe and a SampleJet sample changer. Data were collected using a ^19^F Carr-Purcell-Meiboom-Gill (CPMG) pulse sequence (29, 30) with a total echo time of 160 ms across a sweep width of 126 ppm with an acquisition time of 1 s. All spectra were processed using TopSpin 4.0.9. Fragments were considered binders when the ^19^F signal intensity was significantly reduced in the spectra with protein present compared to the spectra recorded in the absence of protein. The initial screen of 1100 fragments using ^19^F CPMG NMR resulted in 40 fragments that showed binding to the IL-13:VHH204 complex. These were further investigated using ^1^H Saturation Transfer Difference (STD) NMR (31).

STD NMR samples were prepared with a ligand to protein ratio of 100:1 (1 mM ligand, 10 µM protein) in 500 µl 50 mM Tris-HCl, pH 7.5, 100 mM NaCl (90% H_2_O, 10% D_2_O) with 5% d_6_-DMSO to help solubilize the ligand. STD NMR spectra were recorded using a Bruker 600 MHz Avance Neo spectrometer equipped with a 5 mm QCI-F CryoProbe. Data were acquired and processed using the standard Bruker software and collected at 25°C. The protein was saturated in the methyl region of the spectrum at 0 ppm and off-resonance saturation was performed at 33 ppm. A series of 120 50 ms EBurp2 pulses were applied with a 4 µs delay between each pulse resulting in a total saturation time of 6 s. Protein signals were removed by applying a 100 ms spinlock. Interleaved on- and off-resonance data were recorded, processed separately and then the difference spectra obtained by subtracting the on- from the off-resonance spectra. Data were zero filled once and an exponential multiplication window function applied (LB 2 Hz). To rank the relative strength of binding, percentage STD values were determined for each fragment by taking a ratio of the integral area of the peaks in the difference spectrum compared to those in the off-resonance spectrum. Eight hits from the initial ^19^F NMR screen showed a positive response for binding to the IL-13:VHH204 complex by ^1^H STD NMR. These were followed up with protein-observed ^15^N/^1^H TROSY-HSQC chemical shift perturbation mapping.

NMR samples for chemical shift perturbation mapping were prepared with 100 µM of uniformly ^15^N labelled IL-13 mixed with a saturating concentration of unlabelled VHH204 and 1 mM fragment in a 25 mM sodium phosphate, pH 6.0, 100 mM NaCl, 10 μM EDTA, 0.02% w/v sodium azide buffer containing 5% D_2_O/95% H_2_O and 3% d_6_-DMSO. NMR experiments were collected on a Bruker Avance III 600 MHz spectrometer, equipped with a cryoprobe, at 25 °C with acquisition times of 80 ms in ^1^H and 60 ms in ^15^N. Data were processed using Topspin 4.0.5 where the effective acquisition time in ^1^H was cut back to 64 ms. The minimum chemical shift change for each backbone amide peak between free and VHH-bound IL-13 was determined by taking the lowest possible combined shift change value using the following equation:

$\Delta \delta =\frac{\sqrt{{\left(\Delta \delta_{HN}\right)}^{2}+{\left(\Delta \delta_{N}\cdot 0.2\right)}^{2}}}{2}$ where Δ*δ*_HN_ and Δ*δ*_N_ correspond to the difference in ^1^H and ^15^N chemical shifts seen for spectra of the IL-13:VHH204 complex alone and in the presence of fragment hits.

## Results

### Generation of inhibitory VHHs targeting IL-13

**Figure 1:**
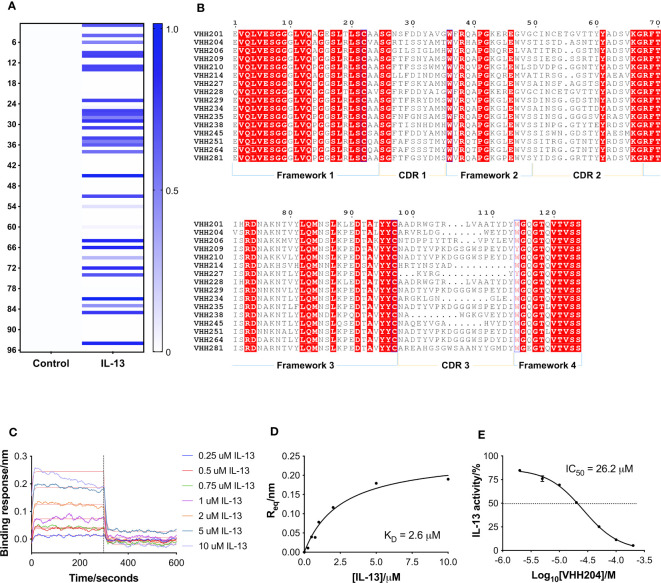
Identification and characterisation of inhibitory VHHs targeting IL-13. **(A)** A heat-map of the monoclonal ELISA results obtained from a naïve library of VHHs biopanned against biotinylated IL-13. The qualitative chart has a binding response from 0.0 to 1.0, where a darker colour does not necessarily correspond to a higher affinity VHH. 30 out of 96 selected VHHs showed binding towards IL-13 (right hand-side). In parallel, the same VHHs were assessed for binding to streptavidin as a control (left hand-side), which showed that all the identified binders are specific for IL-13. **(B)** Multiple sequence alignment of selected representatives of the 16 families of anti-IL-13 VHHs identified. Framework and CDR regions are defined according to Kabat numbering (32). **(C)** Representative BLI sensorgrams are shown for IL-13 binding to VHH204. N-terminally His-tagged VHH204 was loaded onto Ni-NTA biosensors and then titrated with increasing concentrations of untagged IL-13 (0.25 to 10 μM). Double-referenced sensorgrams with corresponding fits (thin red lines) are shown for the 300 s association of IL-13 followed by 300 s dissociation. The highest concentrations of the cytokine (5 and 10 μM) appear to result in increased loss of the VHH from the biosensor, resulting in poorer fits of the association curves. **(D)** Steady-state binding curve (n=1) derived from the sensorgrams displayed in C and fitted with a one-site binding model. **(E)** Dose-response curve (n=2) showing the inhibitory effect of VHH204 on IL-13 signalling in a HEK-Blue cell-based reporter assay. Graphs displayed in **(A, C–E)** were produced using Prism 7 (GraphPad).

A naïve llama VHH phage library, provided by UCB Biopharma, was used to perform phage display biopanning against biotinylated IL-13. Out of 96 VHH clones selected as hits after 2 rounds of panning, a total of 30 showed binding towards IL-13 in a monoclonal ELISA assay (**Figure 1A**). The 30 hits were sequenced by Eurofins Genomics and analysis of the amino acid sequences was carried out using Clustal Omega integrated in the MEGA X software (33). The 30 VHHs were grouped into 16 families, with some differences seen in CDRs 1 and 2, and a greater degree of diversity in CDR3 (**Figure 1B**). VHH families showed diversity in CDR3 in terms of both length (5-19 residues) and biochemical properties, with examples of families with an additional disulphide bond, as well as CDRs with a marked acidic or basic nature. Despite the relatively low number of anti-IL-13 VHHs identified, the VHHs selected through biopanning showed a high degree of sequence diversity (**Figure 1B**).

Following reformatting, bacterial expression and purification of 16 VHHs representative of the families identified, a series of biophysical and functional studies were undertaken. Initially, binding of the VHHs to IL-13 was characterised by bio-layer interferometry (BLI (34)) using an Octet QKe system (Sartorius). His-tagged VHHs were loaded onto Ni-NTA biosensors and titrated with increasing concentrations of untagged IL-13. As illustrated by the example sensorgrams shown for VHH204 binding to IL-13 in **Figure 1C**, IL-13 was confirmed to bind to all the VHHs except VHH206, with a typical concentration-dependent association followed by complete dissociation. Moreover, the steady state binding curves obtained were consistent with a one-site saturation binding model (**Figure 1D** and **Supplementary Figure 1A**), as shown for a number of VHHs representative of distinct sequence families in **Supplementary Figure 1A**. **Table 1** summarises the dissociation constants (K_D_) determined for the panel of anti-IL-13 VHHs, which range from 40 nM to 5.5 μM, as expected for VHHs from a naïve library.

**Table 1:** Affinity and inhibitory activity of a panel of anti-IL-13 VHHs.

VHH	IL-13 K_D_ (µM)	IL-13 IC_50_ (µM)
**201**	0.9 ± 0.1	37.7 ± 1.1
**204**	2.6 ± 0.5	26.2 ± 1.0
**209**	2.2 ± 0.2	67.8 ± 1.0
**210**	0.04 ± 0.008	0.2 ± 0.001
**214**	4.8 ± 0.9	153.8 ± 1.1
**227**	5.5 ± 0.9	63.5 ± 1.1
**228**	0.9 ± 0.1	56.0 ± 1.1
**229**	0.2 ± 0.08	11.4 ± 1.2
**234**	4.1 ± 0.6	49.6 ± 1.1
**235**	3.6 ± 0.3	74.3 ± 1.2
**238**	0.2 ± 0.03	1.7 ± 0.01
**245**	0.2 ± 0.01	3.7 ± 0.01
**251**	2.2 ± 0.5	6.9 ± 1.1
**264**	3.9 ± 0.5	14.5 ± 1.1
**281**	0.4 ± 0.02	34.3 ± 1.1

K_D_ values are reported with the standard error obtained from fitting of the binding curves in Prism. The IC_50_ values are reported as the mean ± standard deviation from two independent experiments.

The effects of the selected VHHs on IL-13 signalling was investigated by an in-cell activity assay using HEK-Blue IL-4/IL-13 Cells (InvivoGen), specifically designed to monitor the activation of the STAT6 pathway induced by IL-13 signalling. IL-13 was pre-incubated with each VHH at a saturating concentration, corresponding to 10 times the K_D_, before addition to the assay. **Figure 1E** shows the dose-response curve obtained for VHH204, revealing inhibition of IL-13 signalling with an IC_50_ of 26.2 ± 1.0 μM. All VHHs showed complete inhibition of IL-13 signalling, with IC_50_ values ranging from 0.2 μM to 153.8 μM (**Supplementary Figure 1B** and **Table 1**). In general, the IC_50_ values determined for the VHHs are in agreement with the corresponding affinities, with a lower concentration of a tight binder and a higher concentration of a weak binder required to fully inhibit IL-13 signalling through IL-13Rα and IL-4Rα.

### Epitope mapping of inhibitory VHHs by NMR

**Figure 2:**
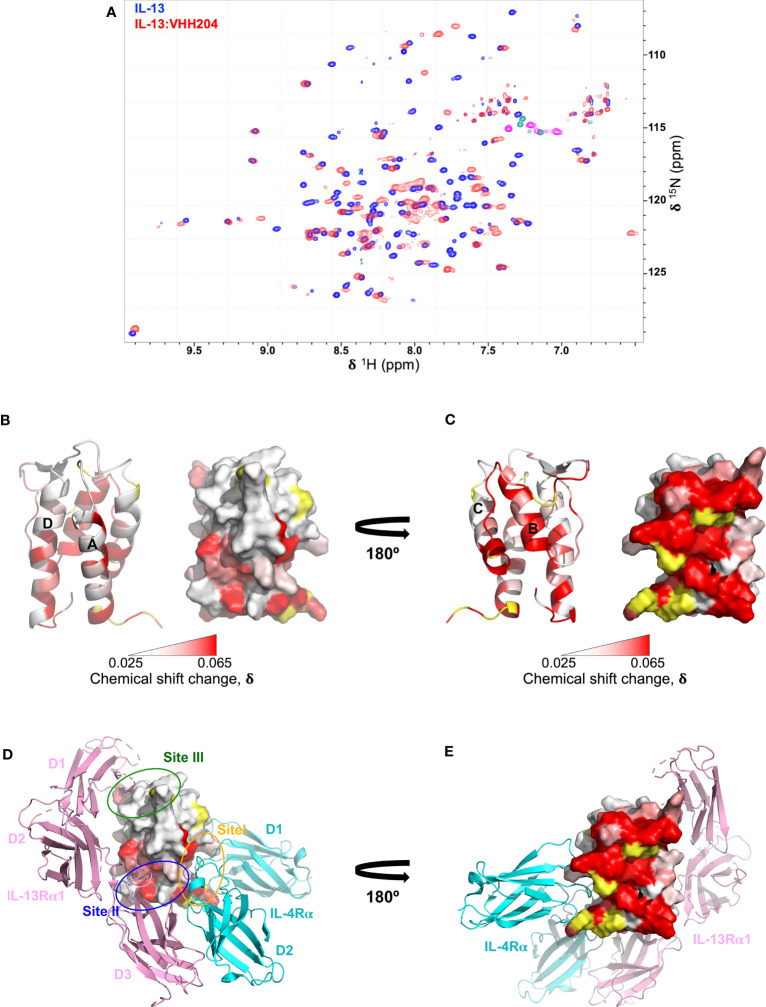
Backbone NMR chemical shift perturbation mapping of the IL-13 binding site for VHH204. **(A)** A contour plot overlay of ^15^N/^1^H TROSY-HSQC spectra acquired from free (blue) and VHH204-bound (red) ^15^N/^13^C-labelled IL-13. **(B, C)** Combined actual and minimal shifts for backbone signals (N, NH and CO) of IL-13 induced by VHH204 binding are highlighted on the structure of IL-13 (PDB: 1IJZ), with equivalent cartoon and surface views shown side by side. A gradient from white to red (0.025 to 0.065 ppm) indicates the size of the chemical shift changes seen, with shifts ≤ 0.025 ppm shown in white and ≥ 0.065 ppm in red. Residues for which no chemical shift change data were obtained are coloured in yellow. The largest backbone chemical shift changes observed clearly form a contiguous patch on the surface of IL-13 formed by helix B and the CD loop, corresponding to the antibody binding site. Minimal shift data was used for residues I37, T40, M43, Y44, A46-I52, F70, C71, H73-S76, G78-F80, L83-R86, which could not be assigned in VHH204-bound IL-13 due to broadening of backbone signals from many residues at the antibody binding site by intermediate exchange. **(D, E)** Surface views of IL-13 in the same orientations as displayed in B and C, with the backbone chemical shift changes induced by VH204 binding shown on the structure of the ternary IL-13 signalling complex (IL-13Rα1:IL-13:IL-4Rα, PDB: 3BPO), which indicates an allosteric mechanism of inhibition. The three domains of IL-13Rα1 are coloured in pink and the two domains of IL-4Rα in cyan. The receptor binding sites on IL-13 are also highlighted by ovals, with Site I (N-terminus of helix A and helix C of IL-13) in orange, Site II (N-terminus of helix A and C-terminus of helix D of IL-13) in blue and Site III (AB loop, CD loop and N-terminus of helix D of IL-13) in green.

We performed NMR chemical shift perturbation mapping studies to determine the binding epitopes for the panel of inhibitory anti-IL-13 VHHs. TROSY-HNCO spectra of uniformly ^15^N/^13^C-labelled IL-13 were collected in the presence of a 10% molar excess of each unlabelled VHH. Minimal backbone chemical shift changes (N, NH, CO) induced by the binding of the VHHs to IL-13 were determined (35) and mapped onto the structure of IL-13, revealing multiple different binding epitopes that decorated the surface of IL-13. These could be grouped into 7 distinct VHH binding sites. The largest group typified by VHH235 contains 8 VHHs, which induce chemical shift perturbations throughout helices A and D, and are likely to sterically block the main interface between IL-13 and IL-13Rα1 (**Supplementary Figures 2B, G, L**). Three VHHs, including VHH227, interact with the site III region of IL-13 and clearly block the binding of IL-13Rα1 domain 1 (**Supplementary Figures 2C, H, M**). VHH245 appears to uniquely bind to the bottom of helices A and D blocking the interaction sites for both IL-13Rα1 and IL-4Rα (**Supplementary Figures 2D, I, N**). The remaining VHH interaction sites identified on IL-13 appear to be inhibitory through non-steric blocking of receptor binding and therefore represent previously unknown allosteric regulatory sites on IL-13. For example, the NMR mapping results for VHH238 highlight that it binds to the C-terminus of helix A and the N-terminus of helix D, which suggests a potential allosteric mechanism of action, or possible steric inhibition through slight overlap with Site I and Site II (**Supplementary Figures 2E, J, O**). Finally, chemical shifts results for VHH204 clearly show that it binds to the long CD loop and helix B of IL-13, which indicates a fully allosteric mechanism of inhibition (**Figures 2B, C**, and **Supplementary Figures 2A, F, K**).

Given the complete inhibition of IL-13 mediated signalling by VHH204, through binding to a novel allosteric site, we chose to study the IL-13:VHH204 complex in more detail. Comprehensive sequence-specific backbone NMR assignments were determined for IL-13 when bound to VHH204. Assignments were made for 72% of the non-proline IL-13 residues, with residues G78-R86 and A46-I52, from the CD loop and helix B, respectively, not assigned as the peaks from many of these residues are missing from 3D spectra of the complex due to exchange broadening. This is consistent with the initial identification of these residues as the region involved in VHH204 binding by NMR minimal shift mapping (**Figure 2A**), and as VHH204 has a K_D_ of 2.6 μM the NMR signals from many residues at the antibody binding site are likely to be in intermediate exchange and substantially broadened/missing (36, 37). The assignment of backbone NMR signals for the majority of residues in IL-13, when bound to VHH204, allowed us to determine the actual NMR chemical shift changes induced by VHH204 binding for residues assigned in both free and VHH204-bound IL-13 spectra. **Figures 2B, C** show the combined actual and minimal backbone chemical shift changes induced by VHH204 binding to IL-13 mapped onto the structure of IL-13, with minimal shift changes shown for residues where no NMR assignments could be obtained for IL-13 bound to VHH204. Substantial chemical shift changes observed for residues within the helical bundle of IL-13 clearly indicate that conformational changes are induced at the receptor binding sites of IL-13 when VHH204 binds to the allosteric site (**Figures 2D, E**).

### Mechanism of inhibition of the allosteric VHH204

**Figure 3:**
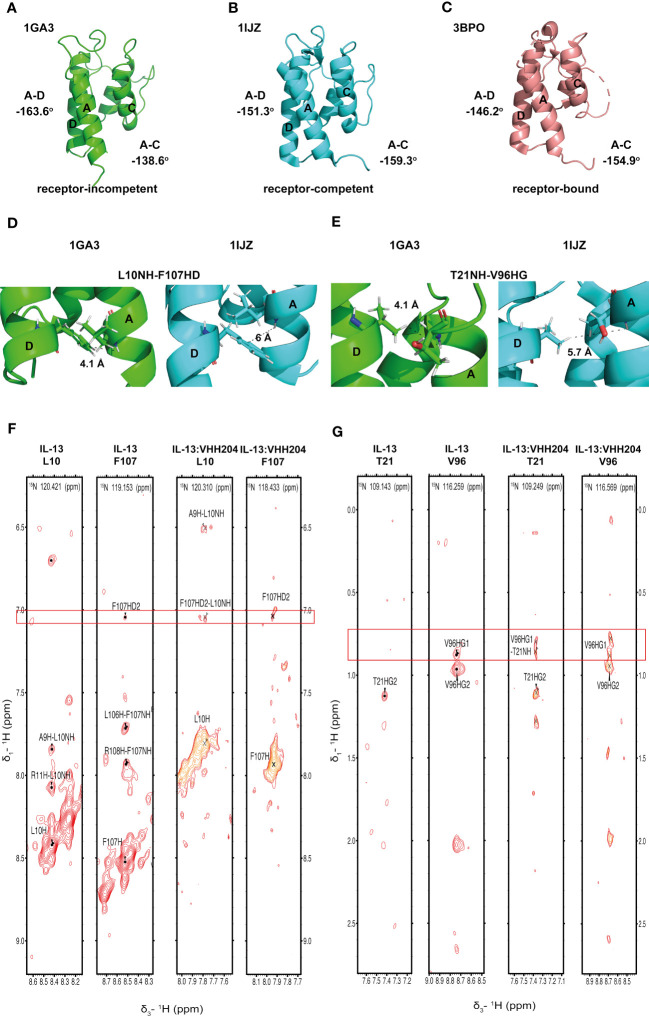
Binding of the allosteric inhibitor VHH204 induces changes in the interhelical angles of IL-13. **(A-C)** The two reported NMR structures of free IL-13 have significantly different interhelical angles. One family of structures has interhelical angles inconsistent with receptor bound IL-13 (A, PDB: 1GA3) whereas the other family of structures has interhelical angles close to those of receptor-bound IL-13 (B, PDB: 1IJZ). For comparison **(C)** shows the x-ray structure of IL-13 solved in complex with its receptors IL-13Rα1 and IL-4Rα (PDB: 3BPO). The substantial changes in interhelical angles seen between the two distinct conformations of IL-13 lead to significant differences in some inter residue distances, resulting in predicted differences in ^1^H-^1^H NOE patterns between IL-13 alone and locked in the receptor-incompetent conformation by bound VHH204. **(D, E)** Examples of backbone amide to side-chain proton distances that differ between the two conformations of IL-13 (L10NH-F107HD and T21NH-V96HG). **(F, G)** Selected backbone amide strips from 3D NOESY-TROSY spectra of both free and VHH204-bound IL-13. Specific inter residue NOEs that were only observed in spectra of VHH204-bound-IL-13 are highlighted with a red box and are consistent with a receptor-incompetent conformation of IL-13.

Several solution structures and associated NMR data have been reported for IL-13 (PDB: 1GA3 [BMRB: 4843], 1IK0 [BMRB: 5004]). We carefully evaluated both the reported IL-13 structures and associated NMR constraints, which revealed that in solution IL-13 exists in two conformations characterised by substantial differences in interhelical angles and the ‘flipping’ of F107 between the surface of the protein and buried within the hydrophobic core (12, 13). The presence of two distinct conformations is reflected in both differences in the reported IL-13 structures and in a significant number of deposited NOE constraints that are not satisfied by a single conformation, such as NOEs involving residues A9, L10 and F107 (13). This conformational heterogeneity for IL-13 probably explains the lack of a reported crystal structure for IL-13 alone. Crystal structures reported for the ternary IL-13 signalling complex show the importance of surface F107 in making contacts with IL-13Rα1 followed by the positioning of IL-13 residues E12 on helix A and R65 on helix C for interaction with IL-4Rα (14). Given that it is known that IL-13 alone has no affinity for IL-4Rα and must first bind IL-13Rα1, we propose that the two solution conformations of IL-13 represent a receptor-incompetent and a receptor-competent state, respectively. More specifically, as determined by QHELIX (38), the receptor-incompetent conformation is characterised by interhelical angles between helices A and D of -163.6° and between helices A and C of -138.6° (**Figure 3A**), while the receptor-competent conformation exhibits A-C and A-D interhelical angles of -151.3° and -159.3°, respectively (**Figure 3B**), in line with the crystal structure of the ternary complex. This latter conformation of free IL-13 is closely comparable to IL-13 bound to its receptors, whereby interhelical angles of -146.2° and -154.9° were calculated between helices A and D and between helices A and C, respectively (**Figure 3C**). It seems likely that binding to IL-13Rα1 stabilises the conformation of IL-13 consistent with binding to IL-4Rα.

Analysis of the combined actual and minimal backbone NMR chemical shift changes induced by VHH204 binding to IL-13 revealed large chemical shift changes for residues at the receptor binding sites on helices A, C and D, including F107 and surrounding residues. Interestingly, residue A9 on helix A showed a large combined backbone chemical shift change of 0.5 ppm. This large upfield shift is possibly due to a shielding effect arising from complete localisation of the F107 side chain in a buried position adjacent to A9, as VHH204 binding stabilises IL-13 in the receptor-incompetent conformation, consistent with the complete inhibition of IL-13 signalling seen in the cell-based assay. To further investigate whether VHH204 was stabilising IL-13 in the receptor-incompetent conformation we characterised the NOE cross-peaks that were visible between backbone NHs and side chain protons in the IL-13:VHH204 complex 3D NOESY-TROSY spectra. Due to differences in the interhelical angles between receptor-competent and receptor-incompetent conformations of IL-13 selected residues will have significantly different backbone amide to side chain proton distances in the two conformations, as illustrated in **Figures 3D, E**, which will be characterised by different NOE cross-peak patterns (39). Representative examples showing the differences in NOE cross-peak patterns observed for residues in free compared to VHH204-bound IL-13 are shown in **Figures 3F, G**. These differences are consistent with binding of VHH204 stabilising IL-13 in the receptor-incompetent conformation. Differences in observed NOE cross-peaks are summarised in **Table 2**. The changes seen in both backbone chemical shifts and NOE patterns associated with specific backbone amides of IL-13 strongly suggest that binding of VHH204 stabilises the receptor-incompetent conformation of IL-13 resulting in the complete inhibition of the cytokine seen.

**Table 2:** Predicted inter residue ^1^H-^1^H NOEs in different conformations of IL-13.

Residue Atoms	Helices	Distance in PDB: 1IJZ (receptor-competent)	Distance in PDB: 1GA3 (receptor-incompetent)	NOE in IL-13:VHH204
**L10NH - F107HD**	A - D	6.0 Å (✗)	4.1 Å (✓)	Yes
**T21NH - V96HG**	A - D	5.7 Å (✗)	4.1 Å (✓)	Yes
**L13NH - M66HE**	A - C	6.2 Å (✗)	4.3 Å (✓)	Yes
**K104NH - L17HD**	A - D	6.9 Å (✗)	3.2 Å (✓)	Yes

For the two distinct conformations of IL-13, selected inter residue distances between protons are marked with a (P) or (O) depending on whether a ^1^H-^1^H NOE cross-peak would be expected in NOE-based NMR spectra, with an expected NOE indicated by a (P).

### ^19^F fragment screen of the IL-13-VHH204 complex

**Figure 4:**
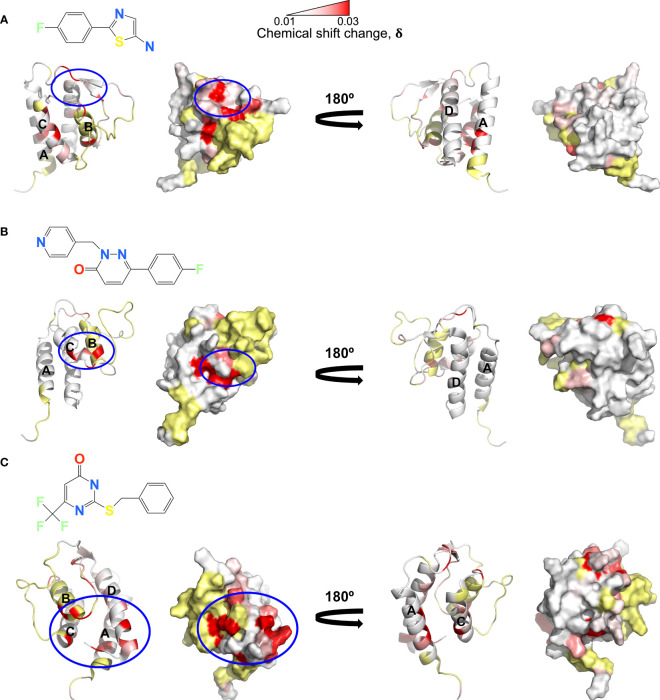
NMR chemical shift perturbation mapping of ^19^F fragments binding to the IL-13:VHH204 complex. **(A–C)** Combined backbone amide (^15^N and ^1^H) minimal shift changes induced by binding of selected ^19^F fragments to IL-13 are shown on equivalent backbone ribbon and surface representations of free IL-13 (PDB: 1GA3). A gradient from white to red (0.01 to 0.03 ppm) indicates the size of the minimal shifts observed, with shifts ≤ 0.01 ppm shown in white and ≥ 0.03 ppm in red. Residues P3-T8, P27, I37, T40, M43, Y44, A46-I52, S58 and C71-V85 for which no minimal shift data were obtained are coloured in yellow. Likely ^19^F fragment binding sites are highlighted by blue circles. Examples are shown for fragment binding sites localised on the AB and CD loop region of IL-13 **(A)**, predominantly on helix C **(B)** and apparently involving the N-terminus of helix B, C-terminus of helix C and adjacent residues on helices A and D of IL-13 **(C)**.

A ^19^F fragment library of ~1100 fragments was screened against the IL-13:VHH204 complex with the aim of identifying hits that bound to the receptor-incompetent conformation of IL-13, which could lead to the development of novel small-molecule inhibitors of IL-13. 40 fragments showed binding to the IL-13:VHH204 complex using ^19^F Carr-Purcell-Meiboom-Gill (CPMG)-based NMR experiments (29, 30) and 8 of these were confirmed to bind to the complex by ^1^H saturation transfer difference (STD) NMR (31). The binding of the 8 confirmed hits was further characterised by ^15^N/^1^H TROSY-HSQC-based chemical shift perturbation mapping of changes induced in uniformly ^15^N labelled IL-13 bound to unlabelled VHH204. Mapping of the backbone amide chemical shift changes induced by the fragments in IL-13 bound to VHH204 resulted in the identification of three different fragment binding sites. The magnitude of the shifts seen were consistent with relatively weak binding, with fragment affinities predicted to be in the micromolar to millimolar range. The first of these binding sites is formed by residues C29-W35 on the AB loop and residues N53-G56 on the BC loop (**Figure 4A**). Interestingly, fragments that bind to this site induce chemical shift changes in several residues with backbone amides orientated towards the interior of the helical bundle, on helices A, D and C. This suggests that small molecules binding to this site have the potential to alter the interhelical angles of IL-13 and potentially act as allosteric modulators of IL-13. The second fragment binding site identified is predominantly made up of residues on helix C (**Figure 4B**). Small molecules binding here have the potential to be functional inhibitors by sterically blocking the interaction with IL-4Rα. The final fragment binding site identified on VHH204-bound IL-13 consists of residues at the N-terminus of helix B and C-terminus of helix C and adjacent residues on helices A and D (**Figure 4C**). Again, the chemical shift changes induced in residues with backbone amides facing into the helical bundle, on helices A, C and D, suggest that small molecules binding to this site could also alter the interhelical angles of IL-13 and have the potential to act as allosteric modulators of IL-13 activity.

## Discussion

Phage display biopanning of a naïve library of llama VHHs, intrinsically characterised by high sequence variability (40), resulted in the identification of a diverse panel of VHHs that bound to IL-13. BLI experiments showed that the panel of VHHs identified exhibited a broad range of affinities for IL-13, with K_D_ values ranging from low nanomolar to low micromolar (**Table 1**), perhaps reflecting the high CDR3 diversity. Cell-based activity assays allowed the effect of each VHH on IL-13 signalling to be evaluated, which revealed that all of the VHHs were inhibitory of IL-13 signalling. NMR chemical shift perturbation mapping studies showed that the panel of anti-IL-13 VHHs bound to a range of different epitopes, decorating the surface of IL-13 (**Supplementary Figure 2**). Interestingly, a number of VHHs were found to bind to previously unknown allosteric sites on IL-13, with the allosteric mechanism of inhibition by VHH204 characterised in detail. The novel allosteric regulatory sites identified on IL-13 could reflect currently unknown mechanisms of regulation *in vivo*.

**Figure 5:**
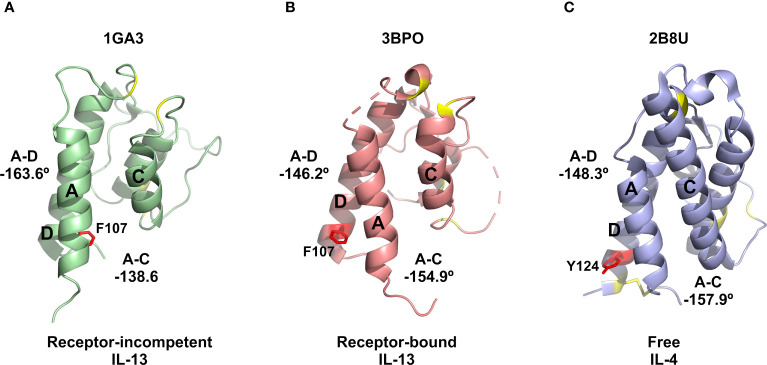
Comparison of IL-13 and IL-4 structures. **(A)** Representative NMR structure of the proposed receptor-incompetent conformation of IL-13 (PDB: 1GA3). **(B)** Crystal structure solved for IL-13 in complex with IL-13Rα1 and IL-4Rα (PDB: 3BPO). **(C)** Crystal structure reported for IL-4 (PDB: 2B8U). In panels **(A, B)** the side chain of residue F107 is shown as red sticks. For comparison, in panel **(C)** the corresponding residue of IL-4 (Y124) is also shown as red sticks. In all panels, the disulphide bonds, including the extra disulphide bond between helices A and D in IL-4, are shown as yellow sticks.

The identification of a number of novel allosteric VHH inhibitors of IL-13 prompted careful evaluation of the two previously reported solution structures of IL-13, together with the associated NMR-derived structural constraints. This analysis revealed that IL-13 exists in two distinct conformations in solution, with one form consistent with the crystal structure of IL-13 reported in complex with its receptors, IL-13Rα1 and IL-4Rα (PDB: 3BPO). The second conformation of free IL-13 shows substantial differences in the interhelical angles between helices A and D (IL-13Rα1 binding site) and helices A and C (IL-4Rα binding site), as shown in **Figure 3A**. Our analysis of the previously reported NMR data and associated IL-13 structures appears to indicate that IL-13 in solution interconverts between these two distinct conformations on a relatively slow timescale (s^-1^). This conformational equilibrium strongly favours the initial binding of IL-13Rα1 to IL-13, resulting in stabilisation of the conformation of IL-13 with interhelical angles consistent with binding to IL-4Rα. This provides a molecular basis for the inability of IL-13 alone to bind IL-4Rα, which requires initial binding of IL-13 to IL-13Rα1 before this binary complex can bind IL-4Rα with a K_D_ of 20 nM (11). In contrast to IL-13, IL-4 binds to IL-4Rα with an affinity of 1 nM, with IL-4 first binding IL-4Rα in the formation of the ternary IL-4 signalling complex (11). Comparison of the deposited structures for IL-13 and IL-4 show several important differences. Firstly, in the reported NMR structures IL-13 residue F107 is shown to flip between a receptor-incompetent buried position (**Figure 5A**) and a receptor-competent surface exposed position (**Figure 5B**). The corresponding residue in IL-4 is Y124. The more polar nature of this side chain would make it energetically less favourable for the side chain of Y124 to be buried in the hydrophobic core of IL-4. IL-4 also has an additional disulphide bridging helices A and D, stabilising the interhelical angle between these helices, together with an extended helix C that could serve to stabilise the orientation of helices A and C (**Figure 5C**). The structural differences between IL-4 and IL-13 further support our hypothesis that a conformational equilibrium in isolated IL-13, involving substantial changes in interhelical angles, is critical in determining the distinct receptor binding selectivity and assembly of the ternary cytokine-receptor signalling complexes.

A structural investigation of IL-13 in complex with VHH204 using NMR showed that VHH204 binds to a previously unknown allosteric site on IL-13, consisting of helix B and a portion of the long CD loop (**Figure 2**). The inhibitory activity of VHH204 (**Figure 1E**) appears to arise from conformational changes induced at the receptor binding sites of IL-13, on helices A, C and D (**Figure 2**). Important differences in long range NOE cross-peaks seen for free and VHH204-bound IL-13 suggest that binding of VHH204 stabilises a conformation of IL-13 with interhelical angles incompatible with binding to IL-13Rα1. To identify small molecules that bind to the receptor-incompetent conformation of IL-13 we screened the IL-13:VHH204 complex against a ^19^F fragment library. Backbone amide NMR chemical shift perturbation mapping of hits from this screen confirmed that a number of ^19^F containing fragments bound relatively weakly to functionally relevant sites on IL-13, or to regions that could potentially induce changes in the interhelical angles of IL-13. This points to novel opportunities to develop small molecule therapeutics targeting IL-13.

To conclude, we have identified and characterised a diverse panel of anti-IL-13 inhibitory VHHs, which has resulted in the identification of several previously unknown allosteric regulatory sites on IL-13. The work reported here has also revealed a novel inhibitory mechanism of action for the allosteric acting VHH204, in which binding to IL-13 prevents any interaction with IL-13Rα1 by stabilising a non-receptor binding conformation of IL-13 present within the conformational equilibrium seen for free IL-13 in solution. This provides new molecular insights into the differing receptor selectivity and assembly of ternary signalling complexes seen for IL-13 and IL-4. We also report the identification of possibly the first small-molecules shown to bind to functionally significant regions of IL-13, which points to the potential to develop small molecule therapeutics targeting IL-13.

## Data availability statement

The original contributions presented in the study are included in the article/**Supplementary Material**. Further inquiries can be directed to the corresponding authors.

## Author contributions

All authors contributed to the design of the experimental work and to analysis/interpretation of the results obtained. The experimental work was carried out by KW, RB, RH, JK, HW, CS, FM, AS-T and CP, with overall supervision of the work provided by AM, AH, AL, GH, CP and MC. The paper was initially written by KW, RB, GH and MC, with all authors contributing to the final version of the text and figures. All authors contributed to the article and approved the submitted version.
