# Supplementary material for: Identification and characterisation of anti-IL-13 inhibitory single domain antibodies provides new insights into receptor selectivity and attractive opportunities for drug discovery
Source: Front Immunol. 2023 Jul 6;14:1216967. doi: 10.3389/fimmu.2023.1216967 (PMC10359924; doi:10.3389/fimmu.2023.1216967)
Supplement: Supplementary file 1 [file DataSheet_1.pdf]

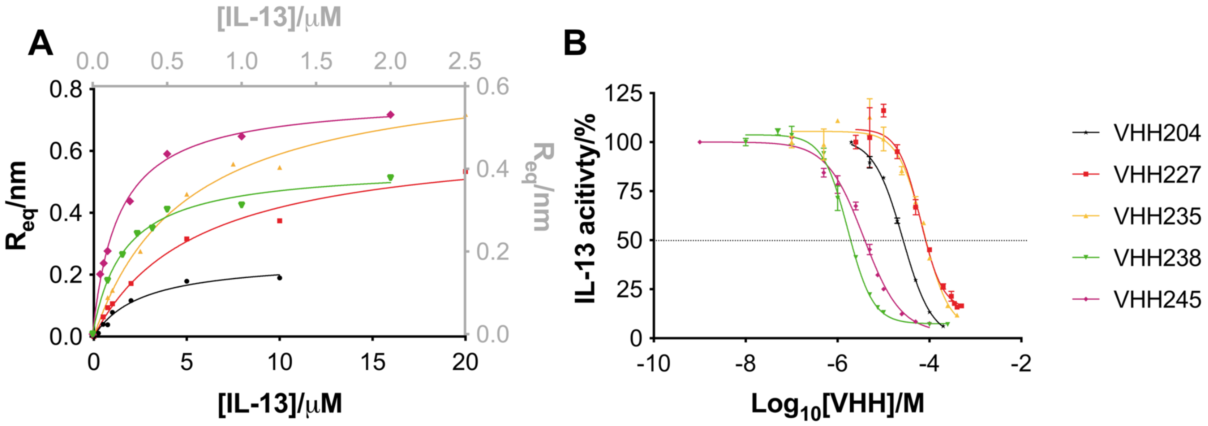

Fig. S1. Binding and functional characterisation of anti-IL-13 VHHs. (A) Steady-state binding curves (n=1) showing the response at equilibrium (R_{eq}) of IL-13 binding to five selected VHHs immobilised on a BLI biosensor, with the curves fitted to a single site binding model in Prism. The selected VHHs shown are VHH204 (black), VHH227 (red), VHH235 (ochre), VHH238 (green) and VHH245 (cyclamen). The black axes refer to VHHs 204, 227 and 235 and the grey axes to VHHs 238 and 245. (B) Dose-response curves (n=2) illustrating the inhibitory effect of the five selected VHHs on IL-13 signalling determined by a HEK-Blue cell-based assay.

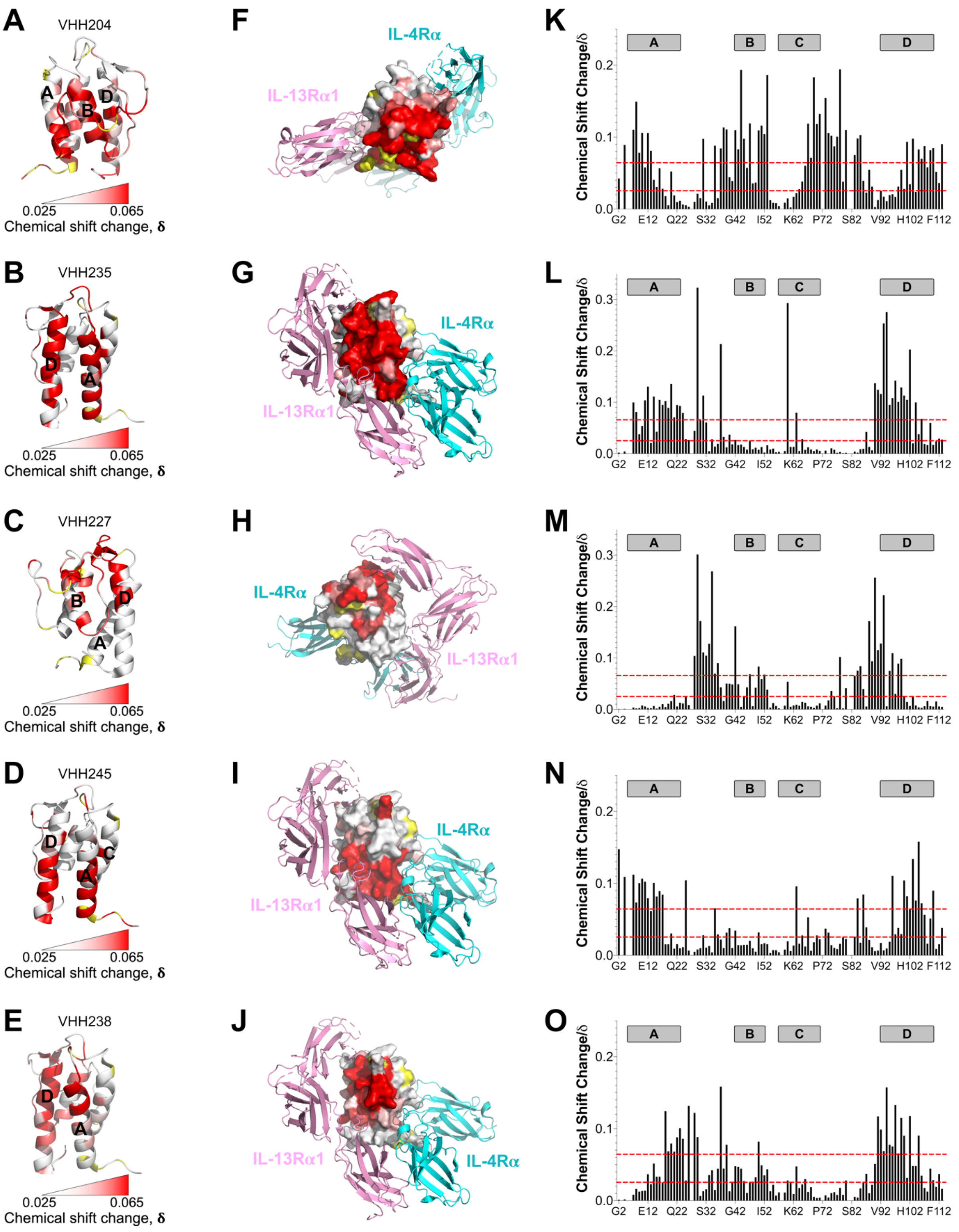

Fig. S2. NMR chemical shift perturbation mapping of VHH binding sites on IL-13. (A-E) Combined backbone minimal shift changes (N, NH and CO) for residues in IL-13 induced by binding of selected VHHs are shown on backbone ribbon representations of IL-13 (PDB: 1IJZ). Residues for which no chemical shift data were obtained are coloured in yellow. A gradient from white to red (0.025 to 0.065 ppm) represents the size of the chemical shift changes induced by VHH binding, with shifts $\leq$ 0.025 ppm shown in white and $\geq$ 0.065 ppm in red. Note that for VHH204 the combined actual and minimal shifts are shown. (F-J) Chemical shift perturbation mapping obtained for the selected VHHs with the ternary IL-13 receptor complex overlaid (PDB: 3BPO). (K-O) Histograms summarising the combined backbone chemical shift changes observed upon VHH binding, with the mapping gradient thresholds shown as a dashed red line. Thresholds were chosen so that the lowest threshold was slightly above the apparent noise and ~15-20 IL-13 residues had shifts greater than the upper threshold, representing the expected number of residues at a VHH binding site. The four helices of IL-13 are shown as grey rectangles above the histogram.
